# Editorial: Core Values and Tasks of Primary Care in Changing Communities and Health Care Systems

**DOI:** 10.3389/fmed.2022.841071

**Published:** 2022-02-07

**Authors:** Johann Agust Sigurdsson, Erika Baum, Rob Dijkstra, Henriëtte Eveline van der Horst

**Affiliations:** ^1^Nordic Federation of General Practice, Reykjavik, Iceland; ^2^Grafarvogur Health Care Center, Reykjavik, Iceland; ^3^General Practice Research Unit, Department of Public Health and Nursing, Norwegian University of Science and Technology (NTNU), Trondheim, Norway; ^4^Department of General Practice, Preventive Medicine and Rehabilitation, Philipps-Universität Marburg, Marburg, Germany; ^5^General Practitioners Training Center, Amsterdam University Medical Center, Amsterdam, Netherlands; ^6^Department of General Practice and Elderly Care Medicine, Amsterdam University Medical Center, Amsterdam, Netherlands

**Keywords:** core values, family medicine, general practice, research topic, history, challenges

## Introduction

Core values of General Practice/Family Medicine (FM) provide a frame of reference for the professional identity and tasks of general practitioners (GPs). They constitute a basis for education, training, professional development and research in FM (1). In a series on core values published in BMJ in 1998, professor in family medicine Ian McWhinney wrote “*Primary care: Core values in a changing world*” (2). Societies are continuously changing in a rapid tempo, and so are healthcare and healthcare systems. Therefore, we continuously need to re-think and re-evaluate our values and tasks.

In line with McWhinney's recommendation, Frontiers in Medicine recently assembled several papers under the Research Topic on core values and tasks of primary care. The Research Topic presents an overview of the topic from a historical perspective of FM, starting with the discipline's establishment in the 1950's, passing significant milestones during the following decades, and currently facing new challenges. Core values in FM in different countries are described, emphasizing topics such as “Continuity of care,” “Person-centered care,” “Comprehensive care,” “Medical generalist care,” “Collaborative care,” “Leadership,” and “Preventive care.”

In the introduction to this Research Topic, we point to major recent or emerging changes, such as aging of the population, an increasing social gradient, “too much medicine” and “superspecialization,” combined with increased commercialization and emergence of “walk-in” centers and opportunistic “on screen doctoring.” These challenges are addressed in the Research Topic's papers by Schmalstieg-Bahr et al. and Sigurdsson et al.

## Different Activities Across Countries

Generally, core values are considered to serve as guiding principles, especially when something important is at stake (2–4). There is however no international consensus regarding the core values of FM/general practice (5). As explained in the Research Topic's paper by Arvidsson et al., activities related to core values in FM differ between countries. In the Nordic countries, England, and Netherlands, the debate on, and subsequent endorsement of, core values has been ongoing for decades [Sigurdsson et al., (6–9)]. In other regions, core values have not yet been discussed and defined. In this context it is interesting to look at the process going on in Ukraine, described by Kolesnyk et al. The authors point out that in a developing country like Ukraine and seen in the light of historical-cultural setting, it is important to harmonize good clinical practice according to the WONCA framework of core competencies with core values.

The Research Topic's paper by Lawson and Nortey further describes the situation in nine African counties, and the authors come to a similar conclusion. In many regions in Africa, FM is not yet a recognized specialty. Nevertheless, FM is gradually expanding. To support the introduction and dissemination of FM in African countries, a clear definition of core values, and particularly of core professional tasks, is of high importance. The methods used by Lawson and Nortey align well with the process of reorientation of core values and core tasks in the Netherlands (7, 8) and the Scandinavian countries [Sigurdsson et al., (1, 9)]. Here, practicing GPs are invited to join the process, starting with more or less open debates, and then narrowing down to define values and tasks that appear most appropriate.

## Research

Substantial body of research and literature confirms the general value of FM as the foundation of any health care system. Well-developed FM is associated with more cost-effective health care and better health (9–11). Some of these studies focus on the core value “Continuity of care.” In 2018, Pereira Gray and co-workers presented a systematic review of studies from nine countries across medical disciplines, concluding that continuity of care was associated with lowered mortality rates (10). In 2021, a study from Norwegian general practice by Sandvik et al. strengthened this conclusion (11).

The core value “Person-Centered care,” is often interlinked with continuity of care. In the core values collection, Burgers et al. present an attempt to systematically review the outcome of person-centered care. They observed that although the idea of person-centeredness might appear evident to a practicing clinician, it is difficult to define and operationalize the concept from a research perspective.

## Education

Studies on education and core values are still few. Therefore, the Research Topic's paper by Michels et al. which addresses the importance of teaching core values is timely and relevant. The authors found that core values are often presented in specific clinical contexts, associated with complex topics such as dementia, multimorbidity and prevention. Mixed teaching and learning methods are often used, combining development of knowledge, skills and attitudes, ideally in small and safe environments. Inspiring role models who demonstrate a holistic view are essential.

Core values can also be taught explicitly. A prospective study from Sudan is promising in this connection. Mohamed et al. analyzed the impact of teaching core values of FM in 2-year Master's programme for training FM physicians in Gezira, Sudan (12). They found that the candidates showed significant progress in most topics related to core values and competencies, including leadership, continuity of care and comprehensive care.

In their Research Topic paper Schmalstieg-Bahr et al. discuss if core values and tasks could be better accomplished by a gatekeeping role for GPs, where GPs act as coordinators of care.

Leadership, one of the core values of our professionalism, can sometimes be motivated by frustration and anger. In the Research Topic's you will find a paper by Heath et al., who encourage GPs not to try and suppress their anger. It is better to use it in a creative manner. Take action, speak truth to power.

In summary, the Research Topic on core values in FM provides an enlightening overview of the role and importance of core values and core tasks in FM. It may inspire FM specialists across the world to assess or (re)define core values in their own country. Historically, the core values were already implicit embedded in the definition of FM from 1950's but they are now more explicit expressed as the disciplines' professional mandate. Thus, although changes in society, organization patterns and standards vary, our core values and principles should be able to endure and unite us.

## Author Contributions

JS made the first draft. All authors: read and approved the final manuscript.

## Conflict of Interest

The authors declare that the research was conducted in the absence of any commercial or financial relationships that could be construed as a potential conflict of interest.

## Publisher's Note

All claims expressed in this article are solely those of the authors and do not necessarily represent those of their affiliated organizations, or those of the publisher, the editors and the reviewers. Any product that may be evaluated in this article, or claim that may be made by its manufacturer, is not guaranteed or endorsed by the publisher.
